# Efficacy and safety of Elaeis guineensis and Ficus deltoidea leaf extracts in adults with pre-diabetes

**DOI:** 10.1186/1475-2891-12-36

**Published:** 2013-04-01

**Authors:** Douglas S Kalman, Howard I Schwartz, Samantha Feldman, Diane R Krieger

**Affiliations:** 1 Nutrition/Endocrinology Department, Miami Research Associates, 6141 Sunset Drive, Suite 301, Miami, FL 33143, USA

**Keywords:** Diabetes, Oral hypoglycemic agents, Phytochemicals, Human studies, Tropical plants, Phenolic compounds

## Abstract

**Background:**

Individuals with pre-diabetes (fasting glucose 100–125 mg/dl) are at increased risk of developing diabetes; 50% of U.S. adults aged ≥65 y had pre-diabetes in 2005–08. Extracts of the leaves of E. guineensis (a tropical plant producing edible oil), and F. deltoidea (a traditional tea) contain phenolic compounds that have hypoglycemic effects in vitro and in vivo. Therefore, a study of the efficacy and safety of these leaf extracts was undertaken.

**Methods:**

Otherwise healthy adults with pre-diabetes (15m/15f; aged 21 to 65 y; BMI ≥25 and < 40 kg/m^2^) were assigned to one of 3 groups for 8 weeks: E. guineensis leaf extract 500 mg or 1000 mg or F. deltoidea leaf extract 1000 mg. Assessments at baseline and throughout the study included: fasting plasma glucose, insulin, OGTT, and HOMA-IR; body weight and waist circumference; vital signs, comprehensive metabolic and lipid panels. Statistical analyses included paired Student’s t-test and ANCOVA or non-parametric tests when indicated.

**Results:**

E. guineensis intervention for 8 weeks decreased fasting plasma glucose and insulin levels, glucose and insulin areas under the curve, and insulin resistance, and increased insulin sensitivity. The 500 mg dose of E. guineensis had a more consistent effect on reducing glycemia than the 1000 mg dose and the insulin findings at the two dose levels were somewhat inconsistent. Differences in the distribution of baseline insulin levels in the low and high dose groups may explain some of these observed differences in responses. F. deltoidea leaf extract had no effect on glycemia variables but both total and LDL cholesterol concentrations were significantly decreased in this group. There were no significant differences in change of weight; however waist circumference was significantly lower in the E. guineensis groups after intervention. At baseline and after 8 weeks of intervention, vital signs and safety lab tests were within normal limits and not significantly different between groups or due to intervention.

**Conclusions:**

These results suggest that the leaf extracts of E. guineensis and F. deltoidea may have positive effects on glucose and lipid levels and are safe for use in humans. Further study is required to determine the maximum effective dosages and the mechanisms of action.

## Background

In 2010, the World Health Organization reported that 347 million people worldwide had diabetes [1], a number that was expected to increase to 439 million adults by 2030. The Centers for Disease Control and Prevention estimated that among U.S. adults aged 65 years and older, 10.9 million had diabetes in 2010 [2]. The increased proportion of people >65 years of age, especially in the U.S, may be an important demographic shift influencing diabetes prevalence [3].

Type 2 diabetes accounts for approximately 90% to 95% of all diagnosed cases of diabetes and is characterized by fasting plasma glucose concentrations (FPG) ≥ 126 mg/dL, 2-hr plasma glucose values (PG) during the oral glucose tolerance test [OGTT] ≥ 200 mg/dL, or hemoglobin A1c values (HgbA1c) ≥ 6.5%. In contrast to the loss of insulin-producing pancreatic beta cells characteristic of type 1 diabetes, type 2 diabetes results from a progressive insulin secretory defect overlaid on a background of insulin resistance [4]. Individuals with pre-diabetes, e.g., those with FPG levels 100–125 mg/dl or impaired glucose tolerance (IGT, 2-h PG values during the OGTT of 140 –199 mg/dl), are at increased risk of developing diabetes, heart disease and stroke. In 2005–2008, 35% of all U.S. adults aged 20 years or older had pre-diabetes, including 50% of adults aged 65 years or older [2]. Extrapolating from these figures to the entire 2010 population suggests that an estimated 79 million American adults 20 years or older currently have pre-diabetes [2].

Intensive lifestyle modification, including a healthy diet, weight loss and physical activity, is the first line of treatment for pre-diabetes because these steps are more effective than medication in reducing diabetes risk [5]. There are currently no medications approved by the U.S. Food and Drug Administration to treat insulin resistance or pre-diabetes [5]. However, the American Diabetes Association recommends that metformin should be considered for use in diabetes prevention, but only for very high-risk individuals who have a BMI of at least 35 kg/m^2^, and are younger than age 60 years [4].

Many alternative therapies — including the anti-inflammatory salsalate [6], rice-containing resistant starch [7], aloe vera [8], and polyphenol-containing compounds such as cinnamon [9] and curcumin [10] — have been suggested to decrease glycemia in individuals with pre-diabetes. Because there have been no long-term trials utilizing these substances, there is no definitive evidence that they are effective; thus, there are no official recommendations for their use.

The oil palm E. guineensis is grown primarily in the tropics and equals soybeans as a source of human vegetable oil consumption [11]. Although the leaf of the oil palm is a waste product, the alcohol extract of the leaf contains large amounts of phenolic compounds [12,13] that reportedly promote vascular relaxation and antioxidant activity in vitro [14]. In a recent study of streptozotocin (STZ)-induced hyperglycemic rats, E. guineensis leaf extract reduced glycemia and lipid oxidation in a dose-dependent manner, possibly by inhibiting dipeptidyl peptidase-4 (DPP-4) secretion [15].

In Malaysia, F. deltoidea has traditionally been taken as tea. Methanol extracts of F. deltoidea plant leaves are rich sources of polyphenolics, flavonoids and tannins, the concentrations of which have been found to correlate with antioxidant activity in vitro [16]. Moreover, in animal studies and cell culture, F. deltoidea leaf extracts were found to enhance insulin-stimulated glucose uptake [17].

Due to the increased prevalence of pre-diabetes and the continuing need for effective pharmacologic treatments of the disorder, the present study was undertaken to test the safety and efficacy of leaf extracts from E. guineensis (OPLE) and F. deltoidea (FICUS) in people with pre-diabetes.

## Methods

The study design was an 8-week, prospective, randomized, and gender-stratified, double-blind, parallel group clinical trial in otherwise healthy adults with pre-diabetes. Subjects included 15 male and 15 female subjects, aged 21 to 65 years, with BMI ≥ 25 and 40 < kg/m^2^, waist circumference greater than 37 inches (94 cm) for males and greater than 31 inches (80 cm) for females, and FPG ≥100 mg/dL (5.6 mmol/L) and ≤ 125 mg/dL (6.9 mmol/L) at the screening visit (Table 1). At baseline, four weeks and eight weeks, primary efficacy was assessed by performing an OGTT to measure fasting plasma glucose and insulin and to calculate HOMA-IR parameters; secondary efficacy was assessed by measuring body weight and waist circumference.

**Table 1:** Baseline demographic and anthropometric characteristics of the subjects

**Intervention**	**OPLE-500**	**OPLE-1000**	**FICUS**	**TOTAL**	**p=**
Enrolled	10	10	10	30	1.0
Completed	9	10	9	28	1.0
Age (y)	44.9±11.4	48.3±13.5	43.5±15.	45.6±13.2	0.72
*Anthropometry					
Weight (kg)	87.2±12.4	91.4±19.3	94.1±14.8	91.0±15.4	0.68
Height (cm)	168±8	168±10	169±14	168±11	0.98
BMI (kg/m^2^)	31.2±4.4	32.1±14.2	33.0±2.2	32.1±3.7	0.59

*Anthropometric variables at the time of screening for study eligibility. Values are the mean ± SD.

There were no significant differences in any of the demographic or anthropometric variables at baseline (Student’s t-test for the continuous variables; Fisher’s Exact Test for the categorical variables).

Safety was assessed at baseline, 2-, 4- and 8- weeks by comprehensive metabolic panel (fasting glucose, BUN, Cr, AST, ALT, ALP, total protein, albumin, globulin, GGT, total bilirubin, calcium, chloride, CO2, sodium, potassium); complete blood count with differential (RBC, WBC, Hgb, Hct, MCV, MCH, MCHC, RDW, Platelets, MPV); lipid panel (total cholesterol, TG, HDL, LDL); blood pressure; heart rate; adverse events; and subjective remarks. Two weeks after beginning intervention, subjects had a brief visit at which vital signs were monitored and blood was collected so that the early safety and efficacy of the products could be determined.

The following test products were provided to the subjects: standardized E. guineensis leaf extract (OPLE; in one of two doses [500 mg, 1000 mg] or standardized F. deltoidea leaf extract (FICUS) (one dose [1000 mg]) produced at Biotropics Malaysia Bhd according to process mentioned in patent: PCT/MY2011/000008. Each capsule of active product contained either 250 mg of OPLE or 250 mg of FICUS. Subjects were instructed to take a total of four capsules per day, two capsules in the morning and two capsules in the evening, with at least eight ounces of water, with or without food, starting the day after being randomized to the study. To maintain the study blind, two placebo capsules were used for the OPLE-500 mg arm. The composition of the phytochemical capsules may be found in Table 2. Subjects were required to bring product bottles to all visits. Compliance was measured via the pill counting method and recorded as a percent of prescribed amount for each visit.

**Table 2:** Composition of the intervention products

**Intervention**	**E. guineensis**	**Ficus**	**Placebo**
Part of Plant Used	Leaves	Leaves	------
Active Ingredients	Oil palm leaf extract	Ficus deltoidea extract	------
Extraction Solvent	50% ethanol, Extraction ratio 10:1	Water Extraction ratio 10:1	------
Inactive Ingredients	Microcrystalline cellulose (100 mg)	Microcrystalline cellulose (100 mg)	Microcrystalline cellulose (280 mg)

Intervention capsules were prepared by Biotropics Malaysia Berhad.

Statistical analyses were carried out as follows: for each continuous variable, the mean change from baseline to each subsequent time point was tested for nominal significance by the paired Student’s t-test or by the non-parametric Wilcoxon test if non-normally distributed. For each continuous variable at each time point, the mean differences in the variable or in the change in that variable from baseline between the different products was tested for nominal significance by the one-way analysis of variance (ANOVA) or by the non-parametric Kruskal-Wallis test if non-normally distributed. For each categorical variable, difference in the distribution of categories between the different product groups was tested for nominal significance by the Fisher Exact test if possible, or by the Chi-Square test if necessary. All p-values appearing in these summarizations are considered descriptive, not inferential. No final statistical conclusions are drawn from them. Fisher Exact tests were generated using the “***R***” statistical/graphical programming system, ver.2.15.0 (***R*** Foundation for Statistical Computing, http://www.r-project.org). AUC for OGTT parameters was determined by trapezoidal integration.

This study was approved by the Aspire Institutional Review Board (Santee, CA) and written informed consent was obtained prior to any study related procedures being performed.

## Results and discussion

Of the 65 subjects who were phone-screened, 47 reported for in-house screening and 31 were deemed eligible for study. Eligible subjects were randomized in a gender-stratified manner, each to one of the three study arms. One subject was lost to follow-up after completing the randomization visit but an additional subject was enrolled and randomized to the same product. One subject dropped out of the study due to an adverse event, leaving 9 subjects in the OPLE-500 group and 10 each in the OPLE-1000 and FICUS groups. There were no statistically significant differences between groups in baseline or demographic characteristics (Table 1) nor were there gender differences of statistical or clinical significance in any of the parameters assessed (data not shown).

**Figure 1:**
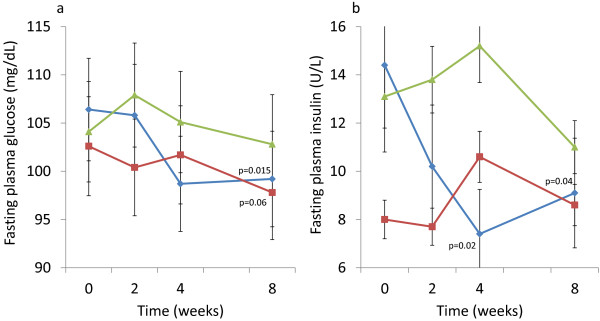
**Change in primary efficacy variables over 8 weeks of intervention with Elaeis guineensis 500 mg/d (−−-**♦**---), Elaeis guineensis 1000 mg/d (−−-**■**---), and Ficus deltoidea 500 mg/d (−−-**▲**---).** Points show the mean value each time point at which the measurement was taken, with vertical error-bars representing ± 1 SD. **a**. Fasting plasma glucose (mg/dL). **b**. Fasting plasma insulin (U/L).

The primary efficacy variables are shown in Table 3 and Figure 1. Final pre-dose FPG values from the OGTT were significantly decreased (p=0.015) in the OPLE-500 group after 8 weeks of intervention compared to the initial pre-dose OGTT FPG values; in the OPLE-1000 group, there was a trend (p=0.06) towards decreased FPG at 8 weeks. Fasting plasma insulin values in the OPLE-500 group were significantly decreased at 4 and 8 weeks (p=0.02; p=0.04, respectively; Figure 1b). There was a non-significant increase in fasting plasma insulin levels at week 4 (+2.6 ± 4.1; p=0.084) with no change at week 8 in the OPLE-1000 group. For subjects in the FICUS group, none of the changes in the primary efficacy variables were found to be significant (Table 3). However, in contrast to the effects of OPLE, there were clinically significant changes in lipids after 8 weeks of intervention in the FICUS group: both total and LDL cholesterol concentrations were significantly decreased (−31 ± 38 mg/dL, p=0.049; and −27 ± 27 mg/dL, p=0.012; respectively).

**Table 3:** Primary efficacy variables after randomization and after 8 weeks of intervention

**Intervention**	**OPLE-500**	**OPLE-1000**	**FICUS**
Fasting Plasma Glucose (mg/dL)			
*Initial	106±11	103±10	104±14
^†^Final	99±10	98±8	106±16
Change	−7.7±7	−5±7	−1±8
p value for change	p=0.02	p=0.06	p=0.61
^₤^Fasting Plasma Insulin (U/ml)			
*Initial	14.4±11.2	8.0±4.3	13.1±5.4
^†^Final	9.1±5.8	8.6±6.1	11.0±6.0
Change	−5.4±7.2	0.6±4.2	−2.1±8.1
p value for change	p=0.04	p=0.96	p=0.14
^₤^Insulin Sensitivity (%)			
^‡^Initial	91 (75)	137 (122)	77 (72)
^‡^Final	172 (123)	136 (119)	114 (93)
%Change	87±113	7±41	91±25
p value for change	p=0.03	p=0.92	p=0.57
^₤^Insulin Resistance			
^‡^Initial	1.66 (1.34)	0.93 (0.83)	1.50 (1.40)
^‡^Final	1.04 (0.82)	0.98 (0.84)	1.27 (1.07)
% Change	−31%±36	−14%±72	−8%±42
p value for change	p=0.055	p=0.92	p=0.65

*Initial values from the baseline OGTT pre-dose sample at the time of randomization to study. Values are given as the mean ± SD.

^†^Final values from the baseline OGTT pre-dose sample after 8 weeks of intervention. Values are given as the mean ± SD.

^‡^Values are given as the mean (geometric mean).

^₤^For each continuous variable at each time point, the mean differences in the variable or in the change in that variable from baseline between the different products was tested for nominal significance by the one-way analysis of variance (ANOVA) or by the non-parametric Kruskal-Wallis test if non-normally distributed. This applied to all of the insulin calculations.

**Figure 2:**
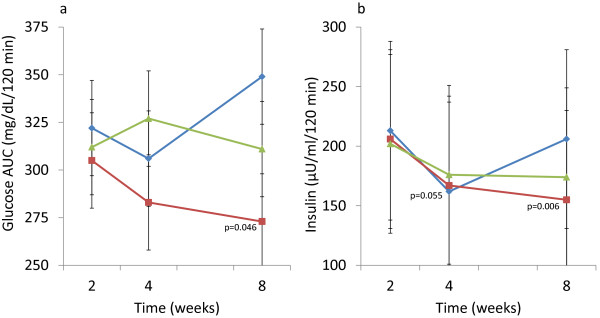
**Change in areas under the curve (AUC) over 8 weeks of intervention with Elaeis guineensis 500 mg/d (−−-**♦**---), Elaeis guineensis 1000 mg/d (−−-**■**---), and Ficus deltoidea 500 mg/d (−−-**▲**---).** Points show the mean value each time point at which the measurement was taken, with vertical error-bars representing ± 1 SD. c. Glucose AUC (mg/dL/120 minutes) d. Insulin AUC (μU/mL/120 minutes).

After 8 weeks of intervention, both the glucose and insulin areas under the curve (AUC) were significantly decreased in the OPLE-1000 group (−32±44, p=0.046; −51±68, p=0.006, respectively; Figure 2a and 2b). Using the homeostatic model assessment estimate of insulin resistance (HOMA-IR), insulin sensitivity significantly increased (87±113%, p=0.027) and insulin resistance decreased (−31±36, p=0.055) in the OPLE-500 group after 8 weeks of intervention The HOMA-IR model is derived from fasting glucose and insulin levels with higher levels representing greater degrees of insulin resistance [18].

**Figure 3:**
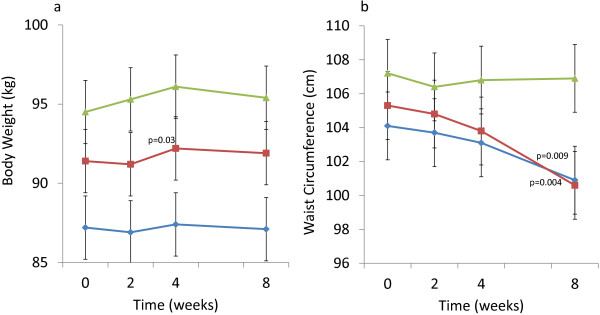
**Change in secondary efficacy variables over 8 weeks of intervention with Elaeis guineensis 500 mg/d (−−-**♦**---), Elaeis guineensis 1000 mg/d (−−-**■**---), and Ficus deltoidea 500 mg/d (−−--**▲**---).** Points show the mean value each time point at which the measurement was taken, with vertical error-bars representing ± 1 SD. **a**. Body weight (kg) **b**. Waist Circumference (cm).

The secondary efficacy variables including body weight and waist circumference were measured at baseline and 4 and 8 weeks after beginning intervention. There were slight increases in body weight of statistical significance at week 4 in the OPLE-1000 group (0.96±1.14 kg, p=0.026) and a trend towards significance in the FICUS group (0.83 ± 1.25 kg, p = 0.080) (Figure 3a). By week 8, however, the magnitude of the weight increases had diminished and there were no significant differences in change of weight compared to baseline in any of the groups (Table 4). There was a non-significant decrease in waist circumference in all groups by week 4. By week 8, however, waist circumference was significantly lower in the OPLE- 500 (−2.8 ± 2.4 cm, p = 0.009) and OPLE-1000 (−4.2 ± 3.5, p = 0.004) groups but not in the FICUS group (−0.4 ± 3.5, p = 0.72; Figure 3b, Table 4.

**Table 4:** Secondary efficacy variables at baseline and after 8 weeks of intervention

**Intervention**	**OPLE- 500**	**OPLE-1000**	**FICUS**
Body Weight (kg)			
*Baseline	87.2±12.4	91.4±19.3	94.5±14.8
^†^Final	87.1±14.1	91.9±19.6	95.4±15.7
Change	0.17±2.04	0.58±2.04	0.10±1.53
p value for change	p=0.81	p=0.40	p=0.41
Waist Circumference (cm)			
*Baseline	104±11	105±11	107±10
^†^Final	101±10	101±11	107±10
Change	−2.8±2.4	−4.2±3.5	−0.4 ±3.5
p value for change	p=0.009	p=0.004	p=0.72

*Baseline body weight is the weight at the time of time of screening for study eligibility.

Values are given as the mean ± SD.

^†^Final body weight after 8 weeks of intervention. Values are given as the mean ± SD.

For each continuous variable at each time point, the mean differences in the variable or in the change in that variable from baseline between the different products was tested for nominal significance by the one-way analysis of variance (ANOVA) or by the non-parametric Kruskal-Wallis test if non-normally distributed.

Safety variables including vital signs, adverse events and safety laboratory data were monitored at screening and weeks 2, 4 and 8. Vital signs were also captured at randomization. At baseline, vital signs and safety lab tests were generally within (or nearly within) normal limits; the few exceptions were not of clinical concern, not outside of eligibility criteria, and not significantly different between groups. After 8 weeks of intervention, there were no significant differences in vital signs or safety lab tests compared to baseline values in any of the intervention groups (data not shown).

No serious adverse events (SAEs) were observed during the course of this study. Eighteen adverse events (AEs) including upper respiratory tract infection, pharyngitis, sinusitis, acne, hematuria, and tooth abscess were observed among 15 of the 30 subjects. One of these events was considered as possibly related to the study product (intermittent light-headedness). One subject dropped out of the study due to an AE, although the event was not considered possibly or probably related to the study product.

Compliance percentages were calculated for weeks 1 through 4 and weeks 5 through 8 and averaged to obtain the overall compliance rate. Overall compliance was greater than 93% for the 8-week period with no significant differences among groups. One subject was not sufficiently compliant with the prescribed amount of product (80% for the first four weeks, and only 50% for the last four weeks, for an overall compliance rate of only 65%). As this subject was in the FICUS group, the final per-protocol population was 28 subjects (OPLE-500 n=9; OPLE-1000 n=10; FICUS n=9).

## Conclusions

This study found a clinically significant, positive effect on fasting plasma glucose levels in individuals with pre-diabetes who were treated with the leaf extract of E. guineensis, a widely grown and utilized tropical palm tree. In addition, waist circumference, an important indicator variable in the metabolic syndrome, decreased in both E. guineensis groups after 8-weeks of intervention. Finally 500 mg and 1000 mg E. guineensis and 500 mg F. deltoidea were found to be safe by all measures utilized.

The 500 mg low dose of E. guineensis appears to have had a more consistent effect on reducing glycemia than the higher 1000 mg dose. Moreover, the insulin findings at the two dose levels were somewhat inconsistent. Differences in the distribution of baseline insulin levels in the low and high dose groups may explain some of these observed differences in responses. It is possible that a larger sample size might result in a less skewed distribution of baseline insulin levels and would result in more similar results in the high and low dose groups.

The HOMA-IR result in the low dose E. guineensis group points to an increase in insulin sensitivity and reduction in insulin resistance as potential mechanisms of action. These findings were not evident in the high dose group which demonstrated an increase in insulin resistance and a compensatory increase in β-cell function. A potential mechanism of action for the improvement in glucose metabolism with E. guineensis involves inhibition of the enzyme dipeptidyl peptidase-4 (DPP-4; [16]) the effect of which is to prevent degradation of gastric inhibitory polypeptide (GIP), which itself stimulates insulin secretion, suppresses glucagon secretion and slows gastric emptying. However, as this study did not assess either DPP-4 activity or the above effects, no comments can be made about this potential mechanism of action.

E. guineensis is rich in catechins and polyphenols [19]. Prior studies in streptozotocin-induced hyperglycemic rats showed that E. guineensis improved proteinuria and reduced oxidative stress levels [20,21]. This suggests a potential benefit for the pre-diabetic and diabetic states. This animal data, coupled with our findings of enhanced glycemic control in subjects with pre-diabetes, indicates that further research in humans is warranted.

Although studies in animals have shown glucose lowering effects with F. deltoidea [16,17], this human study did not support those effects. This study did, however, demonstrate a lipid lowering effect of great clinical interest. The decreases in total cholesterol and LDL observed are of statistical and clinical significance and are worthy of further exploration.

In conclusion, studies of ethanol-derived leaf extracts of the tropical oil palm E. guineensis and the traditional tea F. deltoidea offer opportunities for discovering potential new interventions for pre-diabetes and lipid abnormalities.

## Competing interests

The authors declare that they have no competing interests.

The study was sponsored by Biotropics Malaysia Berhad, Selangor, Malaysia.

## Authors’ contributions

DSK, HIS, SF and DRK all contributed to the study conception, design, acquisition of data and execution. DSK, SF and DRK reviewed the statistical analyses along with the study statistician. All authors had input on the study manuscript. All authors read and approved the final manuscript.

## Authors’ information

DSK, HIS, SF and DRK all work for a contract research organization (Miami Research Associates). DSK is an Adjunct Professor at the Robert Stempel School of Public Health, Florida International University. Both HIS and DRK are Clinical Associate Professors in the Herbert Wortheim College of Medicine, Florida International University, Miami, FL.
